# Assessing of evapotranspiration models using limited climatic data in Southeast Anatolian Project Region of Turkey

**DOI:** 10.7717/peerj.11571

**Published:** 2021-06-09

**Authors:** Yusuf Aydın

**Affiliations:** Nizip Vocational Training School Department of Plant and Animal Production Organic Farming Programme, Gaziantep University, Nizip, Gaziantep, Turkey

**Keywords:** Water consumption, Evapotranspiration models, Climatic data, Hargreaves–Samani equation, Turc equation, Pistachio

## Abstract

Evapotranspiration carries vital importance in areas with arid and semi-arid climate properties for many issues, including the planning of irrigation water as a scarce resource, the establishment of irrigation programs and conducting project design for drainage. The empirical equations used for determining plant water consumption are classified subject to the diversity of the utilized data. The Penman–Monteith method used frequently in many parts of the world as a standard method needs more climate data. Models that yield results that are similar to those of the standard method with less climate parameters are preferred due to their ease of use and wide impact. Temperature, relative humidity and radiation data for the years 2008–2017 were utilized to analyze the usability of the Hargreaves–Samani and Turc-1961 equations with regard to the estimation of reference evapotranspiration in four provinces located in Southeastern Anatolia Region. Results obtained via models were compared in pairs by way of the standard method in order to define the performance of the models. While the best performances were obtained from the comparison with the standard method and Hargreaves–Samani value pair, the comparison of the standard model with Turc displayed the lowest performance. Based on the study data, ET_o-Turc_ values were higher in the provinces analyzed, thus displaying a lower performance. While maximum long term annual monthly average ET_o-HS_ value was identified as 7.6 mm at Diyarbakır in July, whereas the lowest value was determined at Kilis with 5.8 mm; the highest and lowest ET_o-Turc_ values were obtained in the same month at Diyarbakır and Kilis with 13.3 and 10.3 mm respectively. It was calculated based on the long term average annual total ET_o_ values that while highest ET_o-HS_ was calculated at Diyarbakır with 1,500 mm, whereas the lowest value was calculated at Batman with 1,183 mm. The highest value for ET_o-Turc_ was obtained at Diyarbakır with 2,365 mm while Mardin had the lowest ET_o_ value with 1,920 mm. Accordingly, based on the ET_o_ values calculated at both cities studies based on both models, Diyarbakır had the highest values, whereas Kilis had the lowest ET_o_ values. According to the standard method known as PM, lowest daily ET_o_ values were calculated in all provinces, which displayed the highest performance among the models. As a result of this study, it is possible to use the Hargreaves-Samani model instead of the standard model in the absence of reliable climatic data.

## Introduction

Evapotranspiration as “one of the fundamental components of the hydrologic cycle” takes place as a result of the evaporation and transpiration from the soil and leaf surface due to the impact of temperature, radiation, relative humidity and wind speed. On the other hand, evapotranspiration is greatly affected by this change as a biophysical result of the change in land cover. Land cover is an important factor in terms of environmental, ecological, climatic and economic sustainability of the regions as well as the traceability of this change are very important for planning in areas such as climate change and earth sciences. Evapotranspiration had lower values in Northern European countries except Bosnia, Romania, Slovenia and Northern countries of Africa such as Egypt and Libya. On the other hand, areas with low temperature, dense vegetation and high surface soil moisture are more affected by the land-cover change than areas with high temperature, scattered vegetation cover and low surface soil moisture conditions (Li et al., 2021). For this reason, not only climatic parameters are effective in determining ET_o_, but also the topography and spread of vegetation as well as water resources. The amount of water released from the plant and soil due to evapotranspiration leads to an increase in plant water consumption as well as an increase in the need for irrigation. It is of critical importance to estimate evapotranspiration at an optimum level and accurately for regions with high water losses due to evaporation, to utilize the existing resources efficiently due to the necessity to preserve water resources and to increase the use of irrigation technologies that enable limited irrigation applications. Water resources are vital for human life and agricultural production in arid and semi-arid regions. Changes in the levels and amounts of water resources directly affect the quality of life (Congsheng et al., 2020).

Hence, the accurate estimation of ET_o_ is of significant importance, along with studies on the protection and development of water resources as well as irrigation plans (Trajkovic & Stojnic, 2008; Çobaner et al., 2016). Evapotranspiration estimation can be performed by way of direct measurement methods such as field trials, lysimeters (Steduto et al., 1996), Class-A evaporation pans (Maina et al., 2012; Ganji & Kajisa, 2019) in addition to many other empirical equations such as temperature, radiation, mass transfer and combined methods that can be used in different climate and region conditions developed due to the difficulties involved in the implementation of the aforementioned direct measurement methods. For this purpose, Allen et al. (1998) suggested the FAO-56 Penman–Monteith method to enable the more frequent share and use of acquired information and to facilitate ET_o_ prediction. This method is still widely used in the world and is accepted as the standard method. On the other hand, Steduto et al. (1996) reported that even though the developed Penman equations yield highly accurate results in ET_o_ estimation, they display a lower performance compared with the FAO56-PM lysimeter measurements in the Mediterranean Region. However, since the difficulty in obtaining the climate parameters in this method leads to further difficulties in water consumption estimations, Hargreaves–Samani (HS) developed an empirical equality that can calculate the evapotranspiration value with less climate data and based only on temperature and radiation data. Todorovic, Karic & Pereira (2013) suggests the use of “Penman–Monteith Temperature Method-PMT” together with the temperature and radiation based Hargreaves–Samani (HS) equation. Similarly, an equation has been developed by Turc (1961) for the first time for the determination of reference crop water consumption in North Africa and Southern France regions which utilizes only climate parameters such as temperature, radiation and relative humidity. This equation uses only temperature and solar radiation values as data since it is an empirical equation used only for the estimation of reference crop water consumption in humid regions. However, the radiation term in this equation is resolved by the method given in the HS equality.

According to the literature review, studies on plant water consumption in the GAP region where the study was conducted are quite insufficient. The mentioned region has an important place in the geography and agriculture of Turkey. In addition to the main products grown in the region, field crops, pistachio has an important place and has a significant economic value with a share of approximately 93% of pistachio production of Turkey. On the other hand, in the last three decades, with the commissioning of the GAP project, irrigated agriculture has been started in the pistachio fields, and the existing and newly established pistachio orchards have started cultivation under irrigated conditions. Factors such as the fact that Turkey is not a rich country in terms of water, rapid industrialization and urbanization and increasing population made on serious pressures on the demand for food production. Considering that three or four times productivity increase occurs in production with the use of production technologies in irrigated conditions, it is a necessity to develop new water resources and to protect existing water resources in order to meet this production. The GAP region shows terrestrial climate characteristics and the summers are very hot. Low precipitation in the region and irregularity in the precipitation regime are the most important factors that increase evaporation and plant water consumption in summer. Determining easy-to-use ET_o_ models suitable for regional conditions in order to disseminate the studies for determining plant water consumption will enable the efficient use of appropriate irrigation programs and existing water resources. On the other hand, ET_o_ values estimated by ET_o_ prediction models will be multiplied by the K_c_ values determined by FAO-56 on the product basis and will allow the calculation of the actual plant water consumption (ET_c_).

The aim of the present study was to determine the equation which can be used as an alternative to the standard method for the region and which can be utilized with limited climate data. For this purpose, climate data were used for the provinces of Kilis, Diyarbakır, Mardin and Batman located in the Southeastern Anatolia Project region (GAP) and comparative crop water consumption estimates were made through HS and Turc equations utilizing FAO56-PM that is widely used in the world as a standard method. Climatic parameters such as maximum and minimum temperature values, maximum and minimum relative humidity, wind speed, solar radiation were obtained from Meteorological Office. Reference ET_o_ was calculated for all cities using IAM-ETo Software program (Steduto & Snyder, 1998).

## Material & methods

### Geographical location and climate characteristics of the region

**Figure 1:**
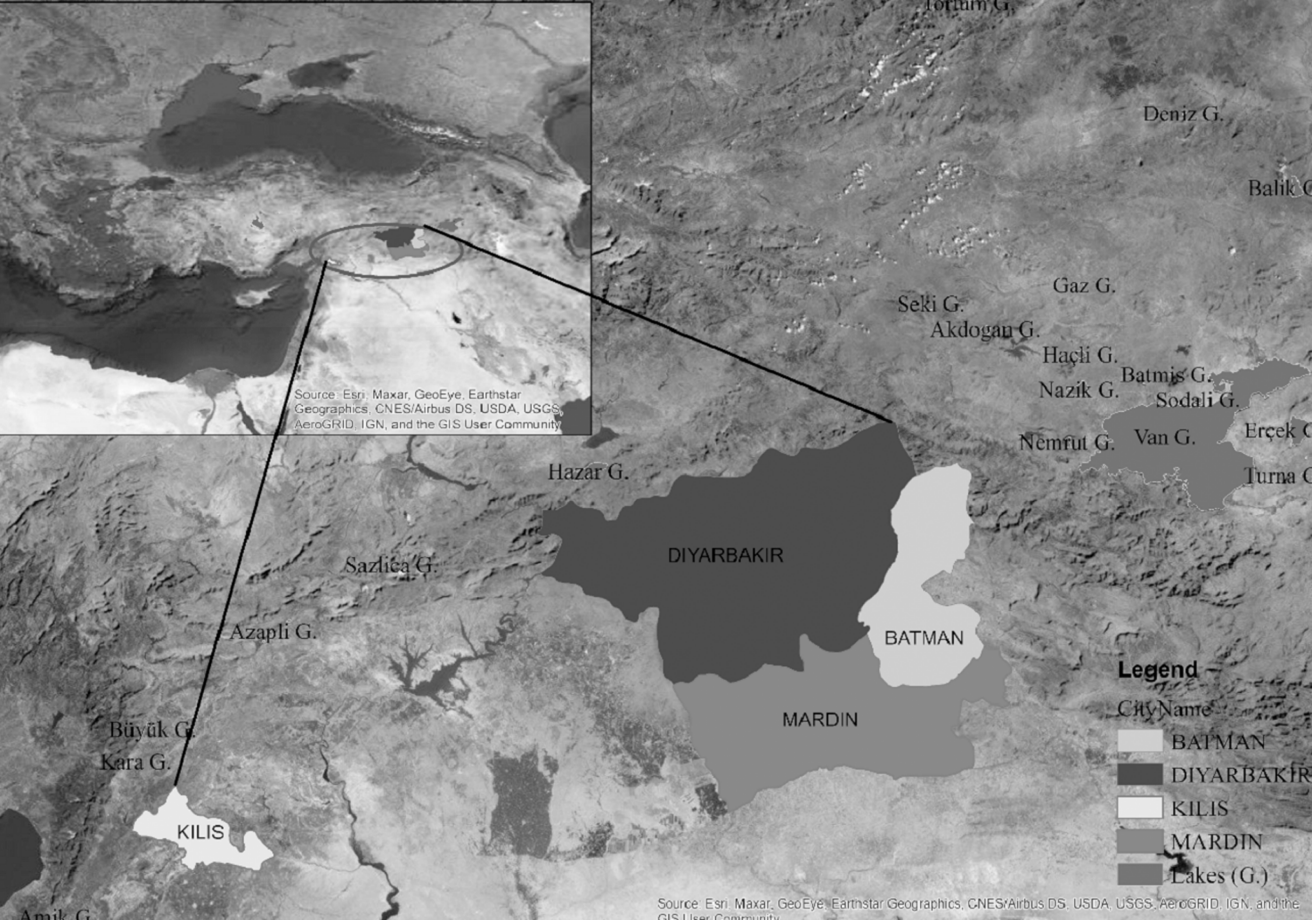
Geographical view of the provinces used in the study.

A large part of the Southeastern Anatolia Region shows terrestrial climate characteristics. The provinces of Kilis, Diyarbakır, Mardin and Batman from Southeastern Turkey were selected for calculating the reference crop water consumption values. Elevations varying between 610–1,040 m and are located at latitudes of 36.7085 to 37.8636 and longitudes of 37.1123 to 41.1562. The climate of the region differs mainly in the Middle Euphrates and Tigris regions. Kilis is located to the west of the region neighboring the Gaziantep province and displays transitional climate characteristics shifting between the Mediterranean and terrestrial climate Whereas the other provinces of Diyarbakır, Mardin and Batman located at the center of the region display continental climate characteristics (Fig. 1). Transition zone characteristics are prevalent due to the Toros mountains located to the west of the region where the Mediterranean climate characteristics decrease and are transformed into terrestrial climate aspects as we move towards the east. Therefore, winter temperature averages are higher than those of the Tigris region. The highest precipitation in this region is observed during the winter season with an average annual precipitation ranging around 700 mm. Terrestrial climate is dominant in the Tigris section with very hot and dry summers and cold and snowy winters. Annual precipitation varies between 500–600 mm. Natural plant cover varies with elevation and low elevations are comprised of plains, weak moorlands and aridity resistant shrubbery (Anonymous, 2021).

Summer months are quite hot in the region with a predominantly continental climate, whereas winters are rarely cold. Hence, average temperature in the month of July during summer is 29.8 °C, while average temperature is 3.7 °C in January which is the coldest month of winter and annual average temperature is 16.4 °C. Even though annual average total rainfall is 565.7 mm, majority of the rainfall takes place in winter and spring months due to irregular rainfall regime. Low relative humidity in the region with an annual average relative humidity of 53.6% results in high evaporation and low rainfall during the summer months leads to arid and long summers in the region (Arıcı et al., 2011). In general, the instability in the precipitation regime of the region leads to less precipitation during the summer months. As a result, the water requirement of plants increases with increasing evaporation values.

**Figure 2:**
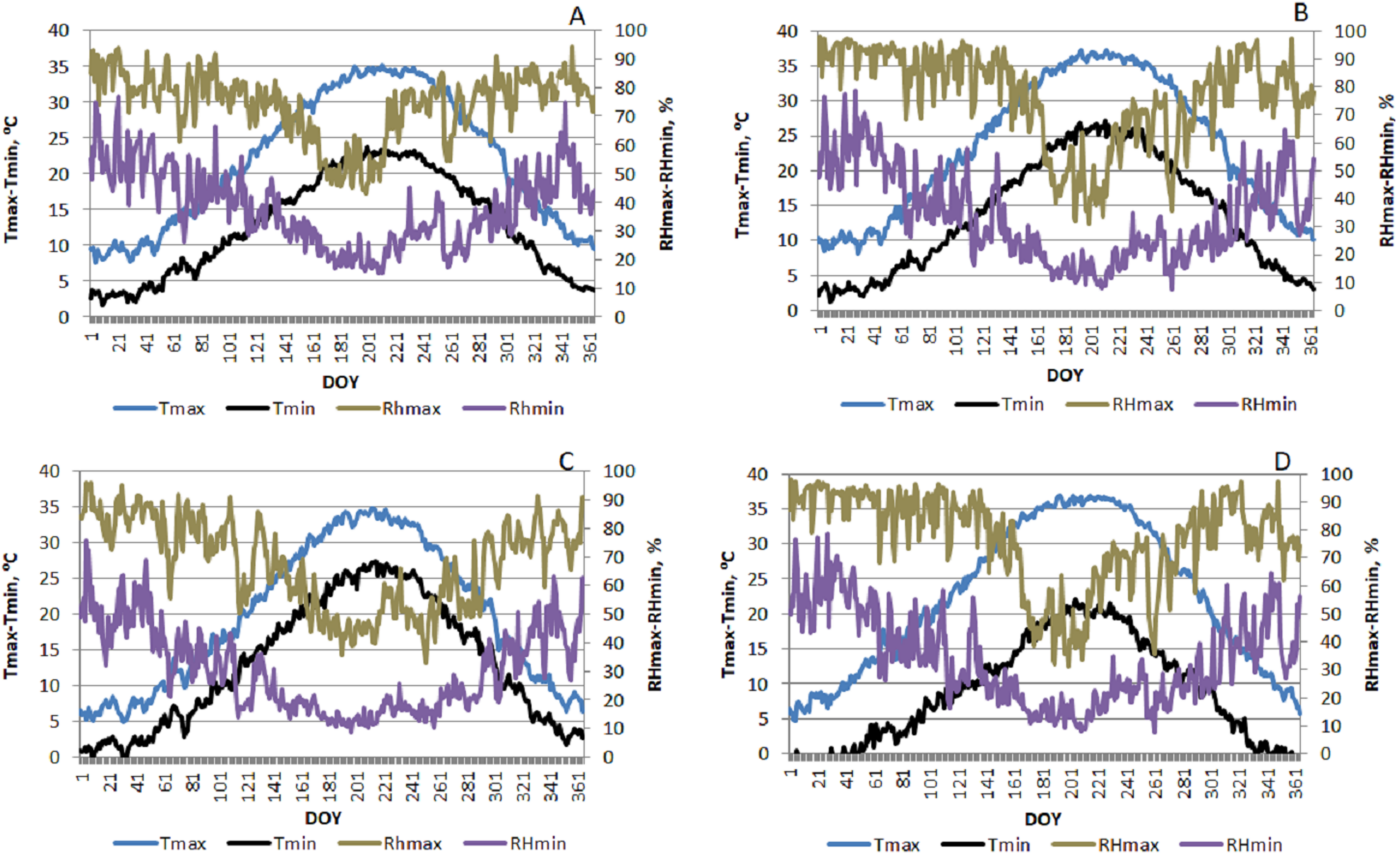
Daily change in the highest and lowest temperature and relative humidity values at the provinces included in the study (2008–2017). A, B, C and D were drawn separately for Kilis, Diyarbakır, Mardin and Batman provinces, respectively, using meteorological records.

Figure 2 presents the highest and lowest temperature and relative humidity values for the provinces included in the region obtained from meteorological records. As can be seen from the graphs, highest temperature values are observed in Diyarbakır from among the provinces included in the study followed by Batman, Kilis and Mardin.

**Figure 3:**
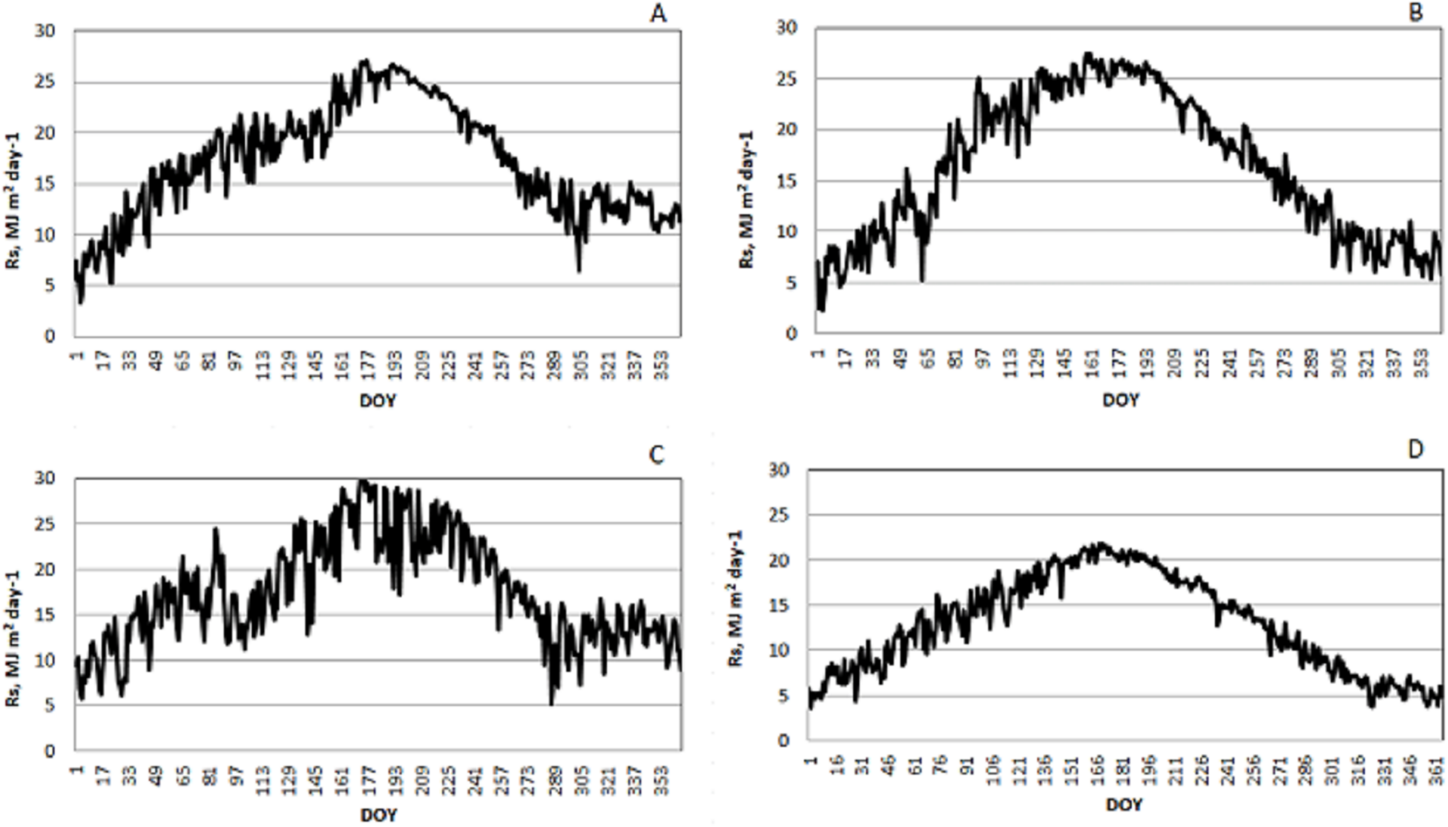
Daily variation of the solar radiation values in the provinces included in the study. A, B, C and D were drawn separately for Kilis, Diyarbakır, Mardin and Batman provinces, respectively, using meteorological records.

Annual change in the solar radiation values obtained from meteorological records has been plotted separately for each city which can be shown in Fig. 3. It can be observed when the plots are examined that the changes in the solar radiation values are similar for Kilis and Diyarbakır, while the R_s-max_ value was determined as 27 MJ m^−2^ day^−1^. These values were 30 MJ m^−2^ day^−1^ and 22 MJ m^−2^ day^−1^ for Kilis and Batman respectively.

#### Methods used to estimate reference evapotranspiration

The FAO56-Penman–Monteith model that is widely used as the standard method for determining the reference crop water consumption is effectively used under conditions in which the climate data can be acquired fully and completely. However, the Hargreaves & Samani (1985) and Turc (1961) models which can operate with limited climate data were used in the present study due to the problems encountered in obtaining climate data and lack of sufficient data. The provinces of Gaziantep, Şanlıurfa, Adıyaman and Siirt that are part of the Southeastern Anatolia Region were selected for analysis in the study by Aydın (2019b) as provinces with extensive pistachio production, whereas in the present study Kilis, Diyarbakır, Mardin and Batman were examined at different time intervals. The climate data for the 2008–2017 period covering 10 years were used in the study. Meteorological records were used to obtain the relative humidity and global radiation data as well as the highest and lowest temperature values used for the calculation of reference crop water consumption (Turkish State Meteorological Service, 2018). Climate data obtained from meteorological records and formulae for the models were imported to the Excel file prepared in accordance with the analysis of reference crop water consumption models after which the reference crop water consumption values were calculated manually. ET_o_ values were evaluated at the annual level following the calculations.

#### FAO56-Penman Monteith method (FAO56-PM)

The Penman–Monteith model is based on evaporation consisting of a grass plant with a height of 0.12 m, surface resistance of 70 s m^−1^ and albedo ratio of 0.23, which can grow well-irrigated conditions without water deficiency thus fully covering the ground surface. As stated in the description of the model, it is generally accepted as the most reliable model in different climatic regions and is widely used globally as the standard method (Shahidian et al., 2012). Thus, the FAO56-Penman Monteith method suggested as the standard method by Allen et al. (1998) for identifying the reference crop water consumption was used to calculate ET_o-PM_. This equation can be written as indicated below (Fisher & Pringle, 2013).

(1) }{}E{T_o} = \displaystyle{{0.408*\left( {{R_n} - G} \right) + \gamma \displaystyle{{900} \over {T + 273}}\; \; {u_2}\left( {{e_s} - {e_a}} \right)} \over {\triangle + \gamma (1 + 0.34{u_2}\; )\; \; \; }}

where; ET_o_: reference evapotranspiration (mm day^−1^); R_n_: net radiation (MJ m^−2^), G: soil heat flux density (MJ m^−2^ day^−1^), T_mean_: average air temperature at 2 m height (°C), U_2_: wind speed at 2 m height (m sn^−1^), e_s_: saturation vapor pressure (kPa), Δ: slope of vapor pressure curve (kPa °C^−1^), γ: psychrometric constant (kPa °C^−1^).

The ET_o_ software developed by FAO56 was used for determining the reference crop water consumption. All parameters used in this software were daily data obtained from the meteorological station.

### Hargreaves–Samani (HS) method

The Penman–Monteith model proposed as a standard method can make predictions based on daily meteorological data. However, the number of meteorology stations that can accurately and reliably measure the climate data required for the model is very limited in many regions of the world and especially in underdeveloped and developing countries. For this reason, models that can operate with less climate data have been developed in climatic regions with no advanced technological tools that can measure parameters such as solar radiation, sunshine and relative humidity accurately. The models developed are classified according to climate parameters which play an active role in ET prediction (Shahidian et al., 2012). The Hargreaves–Samani equation is an empirical radiation-based method used for determining reference evapotranspiration which requires limited weather data such as extraterrestrial radiation (global radiation) (R_a_) and the maximum and minimum (T_max_ − T_min_) temperature values and can be indicated as below (Hargreaves & Samani, 1985).

(2) }{}E{T_{O - HS}} = 0.0023\displaystyle{{{R_a}} \over \lambda }\sqrt {\left( {{T_{max}} - {T_{min}}} \right)} \left( {T + 17.8} \right)

ET_o_: Reference evapotranspiration (mm day^−1^), R_a_: extraterrestrial radiation (mm day^−1^), 0.0023: an ampirical cofficient, T_max_ and T_min_: Maximum and minimum air temperature, λ: the latent heat of vaporization (MJ kg^−1^) for the mean air temperature (T_mean_ in °C) given as:

(3) \lambda = 2.501 - 0.002361*{T_{mean}}

λ is generally assumed 2.45 MJ·kg^−1^

A total of 0.0023 is the empirical coefficient and can also be named as the localization coefficient. Since the equation is determined according to the conditions of the region where it is developed, it should be calibrated when used in different climatic conditions (Shahidian et al., 2012).

Since the temperature, humidity and radiation values used in the equation can be accessed easily, it has been used by many researchers for determining the reference evapotranspiration (Todorovic, Karic & Pereira, 2013; Fisher & Pringle, 2013; Djaman et al., 2015; Çobaner et al., 2016; Diouf et al., 2016; Yamaç, 2018). The R_a_ value used for calculating ET_o-HS_ was calculated using the following equation suggested by Fisher & Pringle (2013) for the Turc method which is solved subject to temperature and by making use of the solar radiation data obtained from the meteorology.

(4) }{}{R_s} = 0.16{\left( {{T_{max}} - {T_{min}}} \right)^{0.5\; }}{R_a}

R_s_ is the solar radiation (MJ m^−2^ day^−1^).

### Turc method

The Turc formula used in this study was developed in France and Northern Africa derived by Turc (1961). This formula is based on some climatic data obtained easily such as air temperature, radiation and relative humidity and therefore easy to apply whenever a full set of climatic data is not easy to collect. This equation for daily potential evapotranspiration can be given as below:

(5) }{}E{T_{o - Turc}} = a*C*\left( {{R_G} + b} \right)\displaystyle{T \over {r + 15}}

where ET_o_: reference evapotranspiration (mm day^−1^), T: mean daily temperature (°C), R_G_: Global radiation (MJ m^−2^ day^−1^), a and b: empirical constants and a = 0.31 (m^2^ MJ^−1^ mm^−1^) and b = 2.094 (MJ m^−2^ day^−1^), C: defined by the relative humidity RH, (%) as:

(6) }{}C = 1 + \displaystyle{{50 - {\rm RH}} \over {70}}\quad if\; RH < 50\%

(7) }{}C = 1\quad if\; RH\; \ge 50\%

Coefficients a and b are provided as empirical coefficients in the original Turc equation and are also known as the localization coefficient. Similarly, the coefficient C given in the Eqs. (6) and (7) is also dependent on the relative humidity. Turc equation has been developed in the humid conditions of France and North Africa. When the specified equation is used in different climatic conditions, it provides overestimate values subject to the Penman–Monteith method (Castañeda & Rao, 2005; Diouf et al., 2016). For this reason, it is recommended that the equation is calibrated according to the climatic conditions in which it will be used.

Similarly, relative humidity and temperature values were obtained from meteorological records of Provincial Directorate of Meteorology in Kilis. The C value in the ET_o-Turc_ equation used for calculating daily potential evapotranspiration was calculated using the aforementioned equations based on the average relative humidity ratio.

### Statistical analysis

In order to further evaluate the compared equations, the equations in Table 1 suggested by Djaman et al. (2015) were used.

**Table 1:** Statistical analysis equations.

Source	Equation
Root mean square error (RMSE	}{}RMSE = \sqrt {\mathop \sum \limits_{k = 0}^n \displaystyle{{{{({P_i} - {O_i})}^2}} \over n}}
Mean absolute error (MAE)	MAE = n^−1^\mathop \sum \nolimits_1^n \left( {{P_i} - {O_i}} \right)
Percentage error (PE)	PE = \left| {\displaystyle{{{P_{ave}} - {O_{ave}}} \over {{O_{ave}}}}} \right|100\%
Mean ratio (MR)	MR = n^−1^\mathop \sum \limits_1^n \displaystyle{{{P_i}} \over {{O_i}}}
Determination coefficient (R^2^) (Todorovic, Karic & Pereira, 2013)	}{}{R^2} = {\left\{ {\displaystyle{{\mathop \sum \nolimits_{i = 1}^n \left( {{O_i} - \bar O} \right).\left( {{P_i} - \bar P} \right)} \over {\sqrt {\mathop \sum \nolimits_{i = 1}^n {{\left( {{O_i} - \bar O} \right)}^2}} .\sqrt {\mathop \sum \nolimits_{i = 1}^n {{\left( {{P_i} - \bar P} \right)}^2}} }}} \right\}^2}

Hargreaves–Samani and Turc equations and the ET_o_ values calculated daily were compared statistically in order to determine the level of correlation between them.

RMSE, MAE, PE and MR parameters were calculated by comparing the ET_o_ values obtained for the provinces included in the study using the models. The daily ET_o_ values calculated for each province were subject to linear regression via paired comparisons for calculating the R^2^ value and thus the R^2^ equation was obtained for the acquired line. The approach to 1 of the R^2^ value used as a criterion for comparing the methods and the approach to zero (0) of the parameters RMSE, MAE, MR and PE presented in Table 1 indicate best performance and point out that there is a strong accordance between the compared values (Fisher & Pringle, 2013; Kaya, Evren & Daşcı, 2016).

## Results

The provinces of Kilis, Diyarbakır, Mardin and Batman located in Southeastern Turkey were selected as regions in the present study. Meterological records of 2008–2017 including maximum and minimum temperature values, relative humidity, precipitation, pan evaporation and solar radiation were used as input data to estimate evapotranspiration (ET_o_) values of the standard daily Penman–Monteith, Hargreaves–Samani (HS) and Turc models.

**Figure 4:**
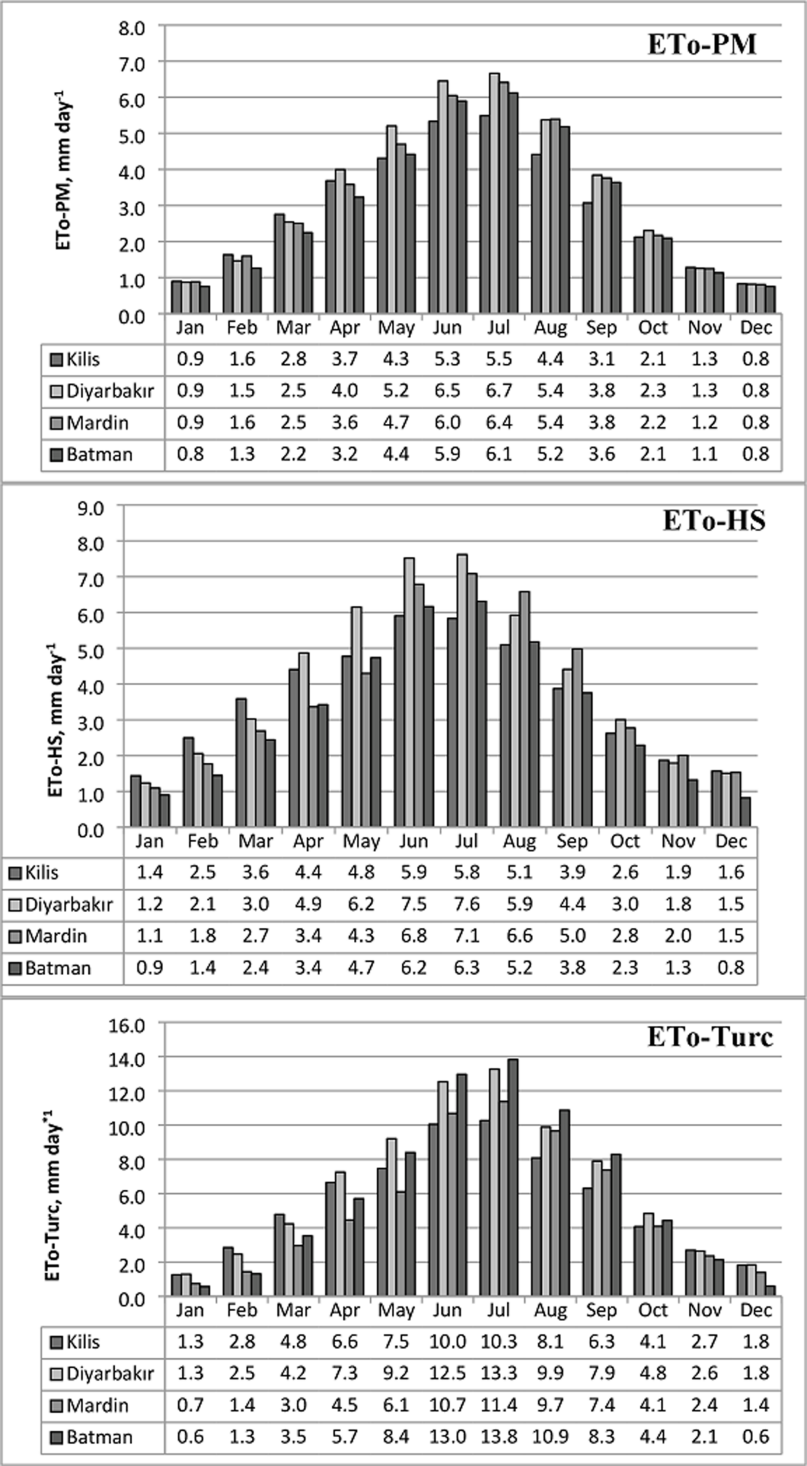
Changes in the long term monthly average values for the province based reference ET_o_ calculated via models (2008–2017). ETo values calculated in daily scale are shown using the models of Penman–Monteith, Hargreaves–Samani and Turc, respectively.

The monthly and annual sums and averages of ET_o_ were calculated by using daily ET_o_ values for each of the year within 2008–2017. The results of each model were presented in Fig. 4 for comparision purpose.

As can be seen from the figure, highest monthly average ET_o_ values were calculated for Diyarbakır followed by Mardin, Batman and Kilis in order. While the highest ET_o-HS_ calculated using Hargreaves–Samani equation for change in the monthly average ET_o_ was obtained as 7.6 mm in July at Diyarbakır, this was followed by Mardin with a value of 7.1 mm. The lowest ET_o-HS_ value was calculated at Kilis with 5.8 mm. The highest ET_o-Turc_ value calculated using the Turc equation was obtained as 13.3 mm at the province of Diyarbakır followed by Mardin with 11.4 mm. Whereas the lowest ET_o-Turc_ value was calculated for Kilis as 10.3 mm. A similar ranking is observed for the change in the monthly average ET_o_ values calculated according to the models and presented in Fig. 4. The ET_o-HS_ values were calculated as much smaller than the ET_o-Turc_ values in all provinces included in the study. This change observed in the ET_o_ values is also present in the changes in the highest and lowest temperatures shown in Fig. 2. The differences observed in the long-term monthly averages were more pronounced in the months when the relative humidity was low and the temperatures were high in the region. During these months, ET_o-HS_ values gave higher values compared to ET_o-PM_, although there were small differences according to provinces. The main reason for the differentiation can be attributed to the variability in temperature and relative humidity values. In the study conducted by Trajkovic (2007) in the hot climatic conditions of Southern Europe, it is stated that the ET_o_ values were estimated with HS are 22% higher than FAO-PM. Similar results reported by Sentelhas, Gillespie & Santos (2010) and Kaya et al. (2017).

On the other hand, it was observed when the ET_o-PM_ values calculated with the standard PM method were examined that lower ET_o_ values have been obtained compared with the other two models. The comparison of ET_o-HS_ and ET_o-Turc_ values with the standard ET_o-PM_, ET_o-HS_ values in all provinces resulted in estimates closer to the standard model.

**Figure 5:**
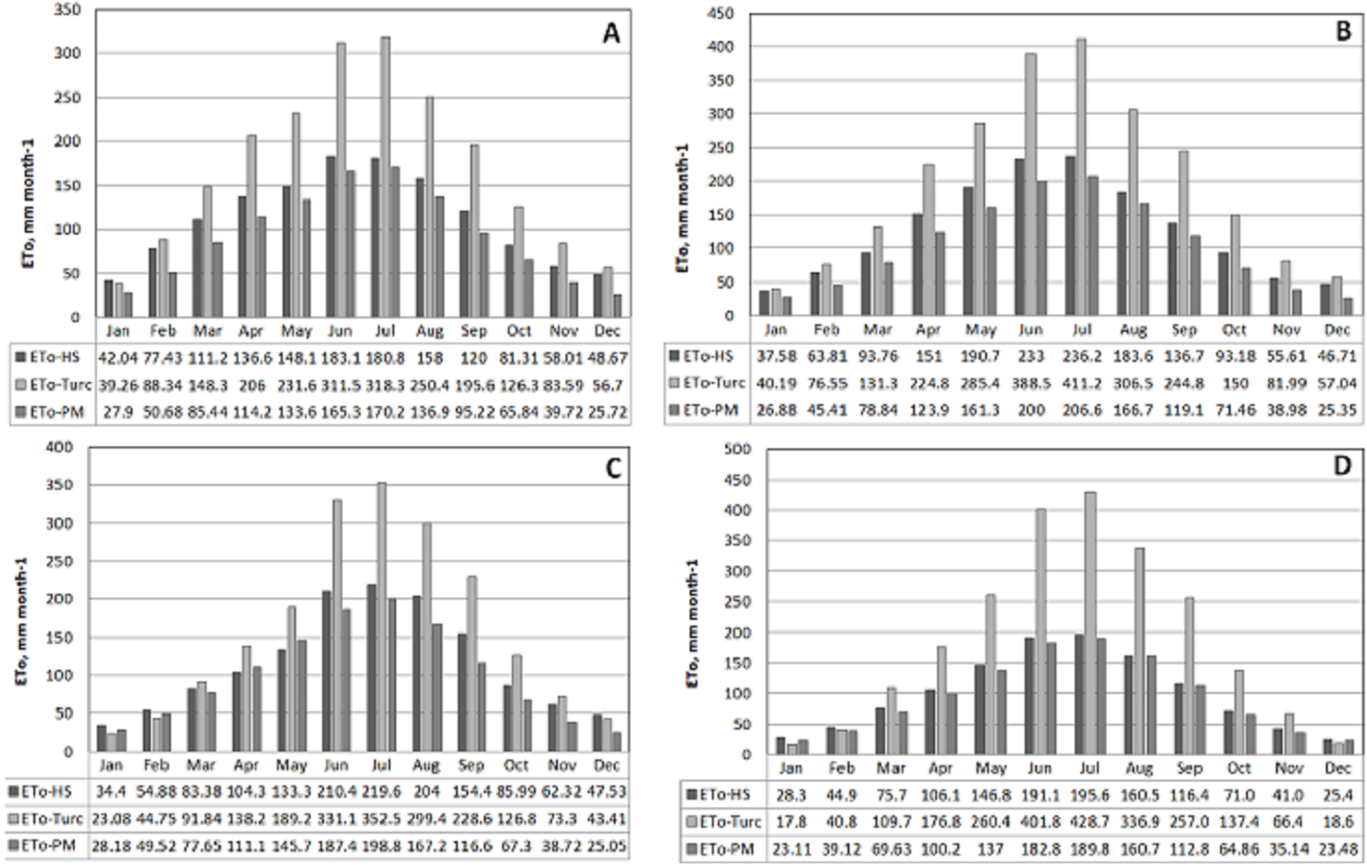
Long term average values for the reference crop monthly total water consumption (2008–2017). A, B, C and D were drawn separately for Kilis, Diyarbakır, Mardin and Batman provinces, respectively, using meteorological records.

**Figure 6:**
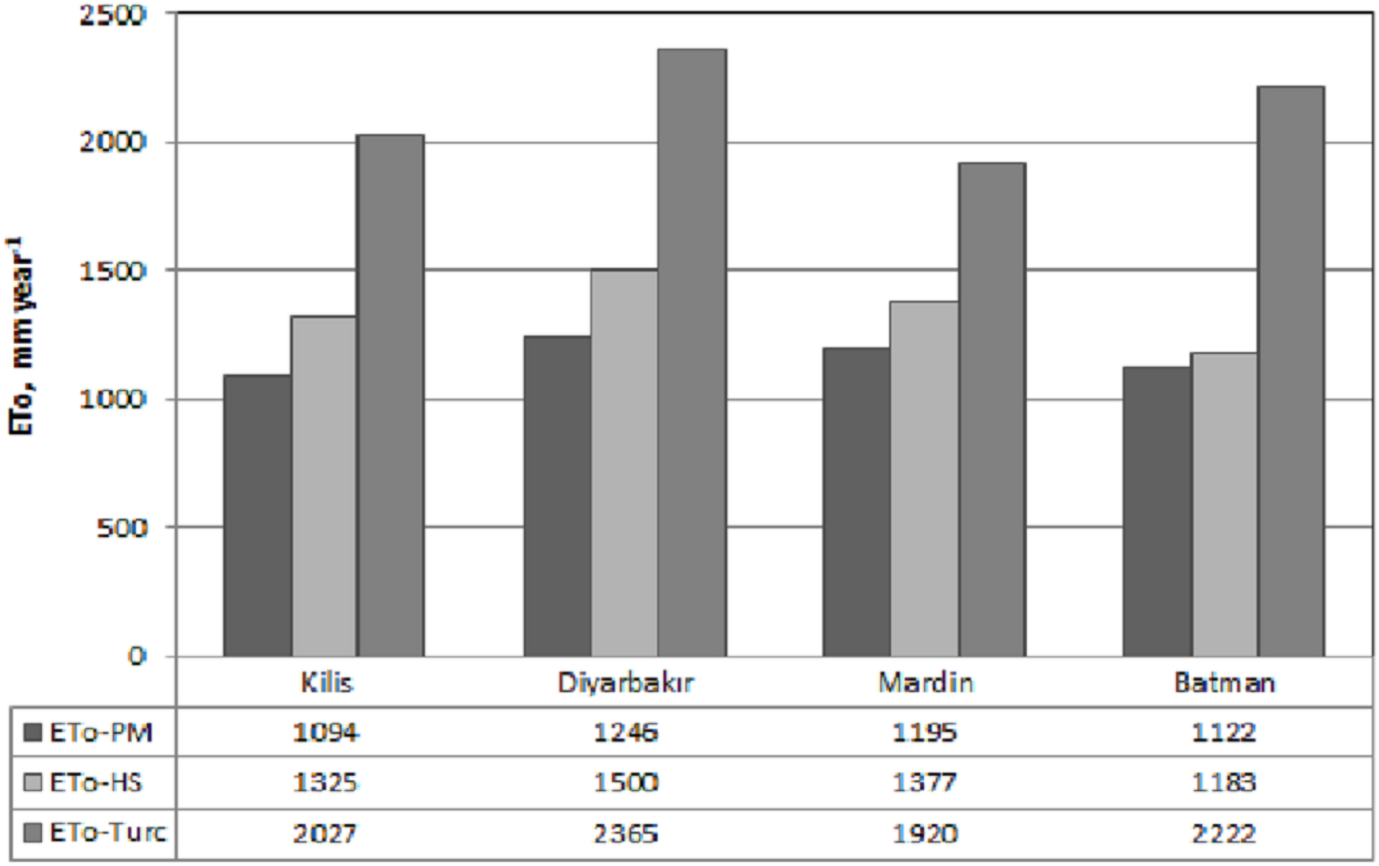
Annual total ET_o_ values for the provinces. The annual totals of the evapotranspiration values calculated on a daily basis with the Penman–Monteith, Hargreaves–Samani and Turc models were compared.

Figure 5 presents the long term average monthly total ET_o_ values calculated separately for the provinces using the equations whereas Fig. 6 shows the long term average annual total.

As can be seen from the graphs, ET_o-PM_ values obtained by the standard method displayed the highest performance by yielding lower values in comparison with the other two models. ET_o-HS_ values were calculated to be lower than ET_o-Turc_ values in all provinces. However, ET_o-HS_ displayed a lower performance compared with ET_o-Turc_ only in the months of January and December in the provinces of Kilis, Mardin and Batman. The monthly total ET_o_ values calculated for the provinces differ while the highest and lowest ET_o_ order was determined for the ET_o_ calculations with both equations as Diyarbakır, Batman, Kilis and Mardin. In general, the PM model displayed the best performance in all provinces compared with HS while Turc model had the lowest performance. The total annual ET_o_ values calculated using the standard model and Hargreaves–Samani and Turc models for all provinces were presented in Fig. 6.

As can be seen in Fig. 6, the lowest ET_o_ values were calculated by the standard PM method, followed by the HS and Turc models. The highest ET_o-Turc_ value was obtained for Diyarbakır with 2,365 mm year^−1^ for the calculation made using ET_o-Turc_ equation whereas the lowest ET_o-Turc_ value was calculated as 1,920 mm year^−1^ for Mardin, while the lowest and highest ET_o_ values obtained during the calculations using ET_o-HS_ equation were again obtained for Diyarbakır and Batman with values ranging between 1,500 mm year^−1^ and 1,183 mm year^−1^. In terms of annual total ET_o_ values, the standard model displayed the highest performance, while the HS model was ranked second followed by the Turc model which displayed the lowest performance.

The ratios of change between the ET_o_ values calculated via equations were determined. Accordingly, This ratio changes between the ET_o-HS_ and ET_o-Turc_ values was calculated as 87.7% for Batman which was followed by Diyarbakır with 57.7%, Kilis with 53% and Mardin with the lowest value of 39.4%. Whereas the average ratio of change for the provinces was calculated as 59.5%. Similarly, highest and lowest change ratios were found in Batman and Kilis as 5.5% and 21.1% respectively in terms of ET_o-PM_–ET_o-HS_. As the average of four provinces the highest ratio of change among models was obtained from the ET_o-PM_–ET_o-Turc_ pair as 83.4% while the lowest ratio was found for ET_o-PM_–ET_o-HS_ as 15.5%. respectively. Thus, in terms of change ratios among models PM-HS pair displayed the best performance while PM-Turc had the lowest performance.

For this reason, the ET_o_ values calculated with the PM model taken as the standard in the study were compared with the ET_o-HS_ and ET_o-Turc_ values calculated using the HS and Turc models. And the acquired results were summarized in Table 2. ET_o_ values were calculated on a daily basis for the presented table.

**Table 2:** Statistical differences of average daily ET_o_ estimated on daily basis as compared to each other.

Provinces	Calculated ET_o_	RMSE (mm d^−1^)	MAE (mm d^−1^)	MR	PE (%)	R^2^
Kilis	ET_o-PM_–ET_o-HS_	0.71	0.64	1.33	21.06	0.9637
	ET_o-PM_–ET_o-TURC_ ET_o-HS_–ET_o-TURC_	2.94 2.42	2.56 1.95	1.87 1.44	85.28 53.04	0.9659 0.9717
Diyarbakır	ET_o-PM_–ET_o-HS_	0.81	0.71	1.30	20.35	0.9719
	ET_o-PM_–ET_o-TURC_ ET_o-HS_–ET_o-TURC_	3.70 3.03	3.07 2.38	1.90 1.49	89.88 57.77	0.9646 0.9761
Mardin	ET_o-PM_–ET_o-HS_	0.80	0.64	1.24	15.21	0.9141
	ET_o-PM_–ET_o-TURC_ ET_o-HS_–ET_o-TURC_	2.79 2.22	2.06 1.63	1.52 1.25	60.63 39.42	0.9031 0.9812
Batman	ET_o-PM_–ET_o-HS_	0.37	0.30	1.09	5.51	0.9727
	ET_o-PM_–ET_o-TURC_ ET_o-HS_–ET_o-TURC_	4.12 3.97	3.16 3.02	1.72 1.60	98.12 87.72	0.9565 0.9650

As can be seen from the Table 2, lowest difference values were obtained from the ET_o-PM_–ET_o-HS_ pair, followed by the ET_o-PM_–ET_o-HS_ value pair when the models were compared with each other in terms of statistical differences. The highest difference was obtained from the ET_o-PM_–ET_o-Turc_ data pair. It was illustrated as a result of the comparison of the provinces included in the study that Batman was ranked first with regard to the ET_o-PM_–ET_o-HS_ value pair of the smallest statistical difference, followed by Kilis, Mardin and Diyarbakır.

The statistical differences vary according to the study provinces. The lowest RMSE value among the provinces included in the study was calculated for ET_o-PM_–ET_o-HS_ value pair as 0.37 in Batman with MAE, MR and PE differences values lower than those for Diyarbakır, Mardin and Kilis. Similarly, the highest RMSE value was also calculated in Batman as 4.12. It was observed when the regression coefficients (R^2^) were examined that the value pairs compared are highly compatible with each other. The R^2^ values of the standard model and the HS and Turc models were generally found to range between 90% and 98% in all provinces included in the study.

However, even though the R^2^ values differ among provinces, the smallest values in terms of statistical differences were obtained for the ET_o-PM_–ET_o-HS_ value pair in all provinces regarding the paired comparisons of HS and Turc models with the standard PM model.

## Discussions

The ET_o_ values calculated using the Hargreaves–Samani equation yielded the closest values to those calculated using the standard method of Penman–Monteith during the study conducted by (Çobaner et al., 2016) for determining the best Hargreaves–Samani equation used for ET_o_ estimation in the Mediterranean Region. The findings of the present study are in accordance with the results of researchers. Trajkovic (2005) indicate that the Hargreaves–Samani equation yields higher results than the actual values in ET_o_ estimation. Regarding the calculation of the reference ET_o_ via Turc-1961 equation, Diouf et al. (2016) carried out a study at Senegal under humid and semi-arid climate conditions during which the ET_o_ values calculated using the FAO56 Penman–Monteith (FAO-PM) and Turc-1961 models were compared. According to the study results: the daily average ET_o_ values calculated via Turc-1961 equation were determined to be lower in arid periods compared with the FAO-PM method, higher predictions were obtained for humid periods. Researchers have indicated that relative humidity is not effective on ET_o_ and that high ET_o_ values are due to temperature and radiation. Aydın (2019a) carried out a study for determining the ET_o_ for the province of Gaziantep using Hargreaves–Samani and Turc equations based on climate data and comparing the results with the standard method. Results closest to those obtained using the Penman model were obtained with the Hargreaves–Samani equation as a result of this study. Turc equation yielded higher ET_o_ values compared with the Hargreaves–Samani equation thus displaying a lower performance.

Based on the assessment of the Southeastern Anatolia Region for the period of 1981–2010, total evaporation amount is indicated as 1,001–1,198 mm year^−1^ (Turkish State Meteorological Service, 2019). The ET_o-HS_ findings obtained in the present study were in accordance with the findings from the provinces of Kilis, Batman and Mardin with higher values in Diyarbakır. Similarly, provinces were ranked as Batman, Kilis and Mardin in terms of the ET_o-HS_ values, Diyarbakır had the highest ET_o-HS_ among the others. However, the ET_o-Turc_ values calculated using Turc-1961 equation are quite greater than those specified in literature. This difference in ET_o_ values between the provinces included in the study is thought to be due to the high temperature values of the provinces and the low relative humidity values during the summer months. Lu et al. (2005) compared three temperature-based methods (Thornthwaite, Hargreaves–Samani, and Hamon) and three radiation-based methods (Turc, Makking, and Priestley–Taylor) for the determination of potential evapotranspiration (PET) in Southern America. The differences between temperature-based methods were observed to be higher compared with those of the radiation-based methods. In general, it has been suggested by Priestley-Taylor that the Turc and Hamon models can be used in regional studies to determine the PET. Yamaç (2018) used the Hargreaves–Samani equation to calculate the reference crop evapotranspiration values in 11 districts of the Konya province where sugar beet is produced. Based on the study results, reference evapotranspiration values were calculated as 1,036 mm year^−1^ and 1,088 mm year^−1^ for Karapınar and Çumra districts. The findings of the present study were greater compared with the findings obtained by the researcher. The difference between these values is considered to be related with the geographical locations of the provinces, climate conditions and the difference between the temperature values.

Statistical assessments using quantitative methods were conducted for identifying the correlation between the ET_o_ values calculated using Hargreaves–Samani and Turc models. Beyazgül, Kayam & Engelsman (2000), Todorovic, Karic & Pereira (2013), Yamaç (2018), Aydın (2019a) reported that the Hargreaves–Samani equation yields the most accurate estimations that are closest to FAO56-PM and also It is recommended to perform local calibration in climatic regions where these equations are used. For this reason, studies have been carried out by many researchers in different climates and countries of the world for the comparison and calibration of models. Some of these can be listed as Arid and semi-arid climatic conditions (Tabari & Talaee, 2011), humid Mediterranean climate (Çobaner et al., 2016; Antonopoulos & Antonopoulos, 2018), humid and arid climatic conditions of Egypt (El Afandi & Abdrabbo, 2015), Iran’s humid climate conditions (Tabari, Grismer & Trajkovic, 2013), in America (Fisher & Pringle, 2013), Brazil (Cabrera et al., 2016), Senegal (Djaman et al., 2015; Diouf et al., 2016), Canada (Xu & Singh, 2001), India (Reddy, 2017). Moeletsi, Walker & Hamandawana (2013) compared the Hargreaves and Samani and theThornthwaite equations in the prediction of evapotranspiration under South African conditions. The utilization of the HS model to predict ET_o_ is suggested in conditions where climate data are not sufficient.

## Conclusions

It was identified as a result of the study that Hargreaves–Samani (HS) equation can be used as an alternative to the standard model for calculating ET_o_ under conditions for which the climate data required for the standard model cannot be obtained. Turc-1961 model yielded very high values for the climate conditions of the region. It is not suggested to use the Turc-1961 model for the development of irrigation programs, prevention of water resources and other hydrologic studies during the reference evapotranspiration studies conducted under the arid and semi-arid conditions of the Southeastern Anatolia Region. It will be possible and easier to determine the actual crop evapotranspiration (ET_c_) using the Hargreaves–Samani model through the use of the crop coefficients (K_c_) of the region for all plants.

## Supplemental Information

10.7717/peerj.11571/supp-1 Supplemental Information 1 Calculation process for KILIS Province.

10.7717/peerj.11571/supp-2 Supplemental Information 2 Calculation process for DİYARBAKIR Province.

10.7717/peerj.11571/supp-3 Supplemental Information 3 Calculation process for MARDİN Province.

10.7717/peerj.11571/supp-4 Supplemental Information 4 Calculation process for BATMAN Province.

10.7717/peerj.11571/supp-5 Supplemental Information 5 Calculation process for KILIS Province.

10.7717/peerj.11571/supp-6 Supplemental Information 6 Calculation process for DIYARBAKIR Province.

10.7717/peerj.11571/supp-7 Supplemental Information 7 Calculation process for MARDIN Province.

10.7717/peerj.11571/supp-8 Supplemental Information 8 Calculation process for BATMAN Province.

10.7717/peerj.11571/supp-9 Supplemental Information 9 Statistical evaluation for study provinces.

10.7717/peerj.11571/supp-10 Supplemental Information 10 Bulk Charts. Figures used in this study collected in one file.

## Additional Information and Declarations

### Data Availability

The following information was supplied regarding data availability: The raw numerical data are available in the Supplemental Files.
